# Editorial: Interactions of microbial biofilms with advanced materials

**DOI:** 10.3389/fmicb.2023.1260753

**Published:** 2023-08-08

**Authors:** Tao Liu, Zhangwei Guo, Shougang Chen, Dake Xu, Tingyue Gu

**Affiliations:** ^1^College of Ocean Science and Engineering, Institute of Marine Materials Science and Engineering, Shanghai Maritime University, Shanghai, China; ^2^College of Materials Science and Technology, Ocean University of China, Qingdao, China; ^3^Shenyang National Laboratory for Materials Science, Northeastern University, Shenyang, China; ^4^Department of Chemical and Biomolecular Engineering, Ohio University, Athens, OH, United States

**Keywords:** biofilm, biofouling, microbiologically influenced corrosion, coatings, nanoparticles, biomineralization, antimicrobial

This Frontiers in Microbiology's Research Topic “*Interactions of microbial biofilms with advanced materials*” under the section “Microbiological Chemistry and Geomicrobiology” presents a total of seven multidisciplinary contributions to highlight state-of-the-start advances in this fast-moving research area. Microbial biofilms on different surfaces have major impacts on various industrial infrastructures and assets, as well as on healthcare. Biofilms can be detrimental because they can corrode or foul various materials and systems, and make infections more difficult to treat. However, they can also be beneficial, for example, by providing corrosion protection via a biomineralization passive layer on a surface. Significant advances have been made in this economically important and highly multidisciplinary scientific field of study in recent years. In nature, microorganisms can influence how materials or chemicals interact with the environment by forming biofilms which are aggregates of sessile cells embedded in exopolymeric substances (EPS) that consist of polysaccharides, proteins and extracellular DNAs. Biofilms provide local ecosystems that can be vastly different from planktonic environments with much higher concentrations of chemicals and the possibly ability to transport extracellular electrons. Their various defense mechanisms make them far more difficult to treat than treating planktonic cells. Physical scrubbing and antimicrobials are traditional approaches in the treatment of problematic biofilms. New methods from different angles are showing promises.

The seven articles under in this Research Topic have been authored by researchers from the United States, China, Sweden and Spain. The Research Topic covers a wide range of areas, such as the development of an analytical tool to distinguish low- and high-biofilm-producing bacterial strains, the use of coatings and antimicrobials against biofouling, microbiologically influenced corrosion (MIC), and biofilm pathogenicity, and many others.

Studies on biocides and coatings that respond against biofouling and MIC to protect metal surfaces are presented in four articles in this Topic. The antimicrobial efficacy and biofilm dispersibility of pH-responsive matrine@chitosan-D-proline (Mat@CS-Pro) nanocapsules are highlighted in the article by Hao et al.. For *Escherichia coli, Staphylococcus aureus*, and *Pseudomonas aeruginosa*, the nanocapsules demonstrated high antibacterial efficacies, reaching 93, 88, and 96% reductions in cell colony counts, against the three bacteria, respectively. The bacteria exposed to the nanocapsules suffered permanent harm with damaged cell structures. After 3 days of incubation, the biofilm thicknesses of *E. coli, S. aureus*, and *P. aeruginosa* dropped by 33, 74, and 42%, respectively, owing to the biofilm dispersal capabilities of the nanocapsules. These nanocapsules were also pH-responsive. They grew to 475 nm diameter at acidic pH. After 10 h of immersion, the matrine concentration released by the nanocapsules reached 28.5 ppm, while the release rate was slower at pH 8.

Conventional polymeric coatings are not suitable for heat exchanger applications against biofilm attachment and MIC, because they are not resistant to erosion, and more importantly, they degrade heat transfer efficiency greatly. The article by Wang et al. describes novel heat-conducting “metallic” coatings with excellent antibiofouling and anti-MIC properties. They demonstrated that NiMo coatings on Ti (used for some heat exchanger tubes) were very effective against a mixed-culture biofilm containing *Chlorella vulgaris* (an alga species) and general heterotrophic bacteria (GHB), as well as a *Desulfovibrio vulgaris* biofilm. After 21 days of incubation, the coating reduced the sessile counts of both *D. vulgaris* and *C. vulgaris* sessile cells by 2-log owing to the presence of CeO_2_ nanoparticles in the NiMo coating. CeO_2_ was found very effective against *D. vulgaris* which is a corrosive sulfate reducing bacterium (SRB), overshadowing the inability of Cu and Ag elements against SRB because of biogenic H_2_S reactivities with them.

The study by Zhang et al. shows how bacterium-induced hierarchically structured minerals on carbon steel surface yielded a multi-functional superhydrophobic coating to protect the metal. In their study, the micro- or nano-scale surface roughness required for superhydrophobic coatings was created via *Bacillus subtilis*-induced mineralization, which was inspired by shell nacre in nature. Hexadecyltrimethoxysilane (HDTMS) was used to cover the biomineralized film, which demonstrated superhydrophobicity with a large water contact angle of 156°. Excellent self-cleaning, anti-icing, and corrosion-resistant performances were displayed by the biomimetic HDTMS/calcite coating. Hierarchically structured biomineralized surfaces at two different length scales, namely a nano-structure roughness to enable water repellency on top of a micro-structure roughness to provide durability, could also be used to produce mechanically robust superhydrophobicity. The approach could serve as a blueprint for the creation of “green” superhydrophobic coatings with multi-functional capabilities in corrosive marine environments to protect various metal surfaces.

In their study, Liu et al. examined the ability of entrectinib, an anti-tumor drug, to inhibit the growth of planktonic and sessile *S. aureus* cells. Their data showed superior antibacterial and anti-biofilm outcomes. Entrectinib also demonstrated a good safety profile, exhibiting no harm against murine 239 T lymphocytes and erythrocytes. Furthermore, in a mouse model of methicillin-resistant *S. aureus* infection, entrectinib decreased the bacterial burden of the septic tissue significantly. A global proteomic analysis of *S. aureus* treated with the drug revealed significant alterations in the expression levels of proteins related to ribosomal structures (*rpmC, rpmD, rplX*, and *rpsT*) and also oxidative stress, suggesting inhibition of bacterial protein synthesis by entrectinib. The fact that entrectinib-treated *S. aureus* produced more reactive oxygen species (ROS) corroborated entrectinib's effect on the expression variations of ROS-correlated proteins associated with the oxidative stress. Moreover, the entrectinib-induced resistant *S. aureus* strain was selected by *in vitro* induction in the presence of the drug and three amino acid mutations. By rupturing the bacterial cell membrane, entrectinib's bactericidal effect on *S. aureus* was verified.

Rodriguez-Temporal et al. assessed matrix-assisted laser desorption ionization time-of-flight mass spectrometry (MALDI-TOF MS) for quantifying biofilms. In 24-h cultures of *S. aureus* and *Candida albicans*, they tested the potential of MALDI-TOF MS to distinguish between the pathogenic microorganism strains that produced low- and high-biofilms. It was found that 38 out of 51 *C. albicans* strains and 95 out of 117 *S. aureus* strains were accurately classified as low- and high-biofilm producers, respectively. Their results suggest that MALDI-TOF MS is an alternative method to traditional crystal violet staining for biofilm quantifications.

The article by Leighton et al. addresses how temperature and strain variations can impact *Vibrio parahaemolyticus* and *Vibrio vulnificus* biofilm formations and their characteristics on glass (GL), low-density polyethylene (LDPE), polypropylene (PP), and polystyrene (PS) surfaces. At 25, 30, and 35°C, every strain of the two bacterial species adhered to GL and every type of plastics. *V. vulnificus* as a species formed a denser biofilm on PS than on GL (*p*-value 0.05), and the biofilm biomass increased at 25°C as opposed to that at 30° (*p*-value 0.01) and 35° (*p*-value 0.02). At the three investigated temperatures, the biofilm biomass and cell density of each strain differed significantly. A positive association (*r* = 0.58) between the dry biomass weight of the biofilm-forming bacterium for each species, and the OD_570_ values obtained from crystal violet staining was revealed. Additionally, it was discovered that both species on all plastics shared the same molecular properties of EPS, with extracellular proteins dominating. The propensity of both species for attaching to plastics was further demonstrated by the hydrophobicity of all the strains at the three temperatures. This research revealed that various *V. parahaemolyticus* and *V. vulnificus* strains quickly produced biofilms *in vitro* with high cell densities on various types of plastics. The biofilm establishment process varied and was species- and strain-specific, and plastic-type dependent, at different temperatures.

In the Research Topic, Yunda et al.'s article studied the formation of methylmercury (MeHg) in *Geobacter sulfurreducens* biofilms. MeHg is the methylation product of divalent inorganic mercury that that accumulates in the biota. Biofilms cultured in the culture medium with vitamins for 3 days showed the largest surface coverage, and they secreted ample amounts of MeHg in the biofilm EPS. Using 3- and 7-day biofilms, the authors demonstrated that *G. sulfurreducens* biofilms cultured with different nutrient loads produced MeHg, much of which released into the surrounding medium. In a low-nutrient medium with a 3-day biofilm, the mercury methylation rate constant measured in a 6-h assay was 3.9 ± 2.0 × 10^−14^ L · cell^−1^ · h^−1^, which is 3X to 5X lower than the rates measured in this and previous studies with sulfur reducing *G. sulfurreducens* planktonic cultures. However, the proportion of MeHg in the biofilms was as high as almost 50% in total mercury, and inorganic mercury exhibited only low levels of accumulation in the biofilms. These findings suggest that the *G. sulfurreducens* biofilms had a high ability of mercury methylation, and the transfer of Hg(II) to the *G. sulfurreducens* biofilm was the rate-limiting step of MeHg formation in this system.

In summary, this Research Topic describes several new developments, involving bio-scientists, medical researchers, and corrosion and materials engineers, that provide a glimpse of the exciting biofilm research field. Some of the studies are preliminary studies that may inspire further studies, leading to potential field applications.

## Author contributions

TL: Writing—original draft, Writing—review and editing. ZG: Writing—original draft. SC: Writing—review and editing. DX: Writing—review and editing. TG: Writing—original draft.

